# Mining the Microbiome of Key Species from African Savanna Woodlands: Potential for Soil Health Improvement and Plant Growth Promotion

**DOI:** 10.3390/microorganisms8091291

**Published:** 2020-08-24

**Authors:** Ivete Sandra Maquia, Paula Fareleira, Isabel Videira e Castro, Denise R. A. Brito, Ricardo Soares, Aniceto Chaúque, M. Manuela Ferreira-Pinto, Erica Lumini, Andrea Berruti, Natasha S. Ribeiro, Isabel Marques, Ana I. Ribeiro-Barros

**Affiliations:** 1 Plant Stress & Biodiversity Lab—Forest Research Center (CEF), School of Agriculture, University of Lisbon, 1349-017 Lisbon, Portugal; ivete.s.maquia@uem.ac.mz (I.S.M.); manuelafpinto@isa.ulisboa.pt (M.M.F.-P.); 2 TropiKMan Doctoral Program, Nova School of Business & Economics (Nova SBE), 2775-405 Carcavelos, Portugal; 3 Biotechnology Center, Eduardo Mondlane University, CP 257 Maputo, Mozambique; nisebrito@gmail.com; 4 Instituto Nacional de Investigação Agrária e Veterinária, I.P. (INIAV, I.P.), 2780-159 Oeiras, Portugal; paula.fareleira@iniav.pt (P.F.); isabel.castro@iniav.pt (I.V.eC.); ricardo.soares@iniav.pt (R.S.); 5 Faculty of Agronomy and Forest Engineering, Eduardo Mondlane University, CP 257 Maputo, Mozambique; achauque2012@gmail.com (A.C.); nribeiro@uem.mz (N.S.R.); 6 Institute for Sustainable Plant Protection, National Research Council, I-10135 Turin, Italy; erica.lumini@ipsp.cnr.it (E.L.); berruti.andrea@gmail.com (A.B.)

**Keywords:** 16SrRNA, fire, Limpopo National Park, plant growth-promoting bacteria, rhizosphere, Mopane, Combretum

## Abstract

(1) Aims: Assessing bacterial diversity and plant-growth-promoting functions in the rhizosphere of the native African trees *Colophospermum mopane* and *Combretum apiculatum* in three landscapes of the Limpopo National Park (Mozambique), subjected to two fire regimes. (2) Methods: Bacterial communities were identified through Illumina Miseq sequencing of the 16S rRNA gene amplicons, followed by culture dependent methods to isolate plant growth-promoting bacteria (PGPB). Plant growth-promoting traits of the cultivable bacterial fraction were further analyzed. To screen for the presence of nitrogen-fixing bacteria, the promiscuous tropical legume *Vigna unguiculata* was used as a trap host. The taxonomy of all purified isolates was genetically verified by 16S rRNA gene Sanger sequencing. (3) Results: Bacterial community results indicated that fire did not drive major changes in bacterial abundance. However, culture-dependent methods allowed the differentiation of bacterial communities between the sampled sites, which were particularly enriched in Proteobacteria with a wide range of plant-beneficial traits, such as plant protection, plant nutrition, and plant growth. *Bradyrhizobium* was the most frequent symbiotic bacteria trapped in cowpea nodules coexisting with other endophytic bacteria. (4) Conclusion: Although the global analysis did not show significant differences between landscapes or sites with different fire regimes, probably due to the fast recovery of bacterial communities, the isolation of PGPB suggests that the rhizosphere bacteria are driven by the plant species, soil type, and fire regime, and are potentially associated with a wide range of agricultural, environmental, and industrial applications. Thus, the rhizosphere of African savannah ecosystems seems to be an untapped source of bacterial species and strains that should be further exploited for bio-based solutions.

## 1. Introduction

Forest soils have some of the most complex microbial communities on Earth [1,2] which are among the major ecological determinants of those ecosystems [3]. Emerging studies highlight the importance of microbial applications, particularly plant growth-promoting (PGP) rhizobacteria, in the context of sustainable agricultural systems [4,5,6,7,8]. Bacteria belonging to the phyla Proteobacteria, Actinobacteria, Acidobacteria, Bacteroidetes, Chloroflexi, Firmicutes, Gemmatimonadetes, Planctomycetes, and Verrucomicrobia have been reported worldwide. Their diversity depends on factors such as temperature, pH, soil type, vegetation, and land use [9].

Africa constitutes a wealth of global biodiversity distributed over a wide range of different ecosystems [10]. Among them, the Mopane woodlands are considered the second most important ecoregion of Sub-Saharan Africa due to its ecological, cultural, and socioeconomic value [11,12], covering approximately 555,000 km^2^ distributed across northern Namibia, southern Angola, central Malawi, central and southern Mozambique, northern South Africa, and throughout Zimbabwe, Botswana, and Zambia [13,14]. This ecosystem is usually characterized by the dominance of monospecific stands of the legume tree *Colosphospermum mopane* (J.Kirk ex Benth.), although it is also associated with several other prominent trees and shrubs, such as the red bush willow *Combretum apiculatum* Sond. [15].

Mopane woodlands are frequently exposed to human and climate pressures, which in association with herbivory and grazing, are intimately associated with the occurrence of fires [16]. Altogether, these factors may affect the vegetation structure and composition, and thus the ecosystem’s stability [17,18]. Therefore, analyzing the history of fire regimes, as well as species’ adaptation to fire, is essential to understand key ecosystem processes and biodiversity dynamics for decision-making support [15,19]. However, studies that elucidate the interactions of African vegetation with ecological drivers are recent and still scarce [15,20]. Ribeiro et al. [15] reported that while the current fire regimes in the Limpopo National Park (LNP, Mozambique) do not have a negative impact on the vegetation structure or composition, long-term fire frequencies may impose key changes on biodiversity dynamics. On the other hand, the work of Burbano et al. [20] in the Kunene region of Namibia was the first extensive report on the diversity and structure of nitrogen-fixing bacteria associated with *C. mopane*, highlighting an outstanding diversity of PGP diazotrophic bacteria in legume tree roots, many of which are as yet uncultivated, as well as the difficulties in detecting symbiotic root nodules.

In this work, we have characterized the richness and diversity of rhizobacteria in the mopane woodlands of LNP, namely in the rhizosphere of the dominant tree legume *C. mopane* and the subdominant non-legume tree *C. apiculatum,* in three different landscapes subjected to two different fire regimes. For this, 16S rRNA high-throughput sequencing was performed, followed by the isolation of PGP bacteria, i.e., diazotrophs, phosphate solubilizers, hormones, and siderophore producers. The presence of the symbiotic diazotrophs was further characterized using the tropical generalist legume *Vigna unguiculata* (L.) Walp to obtain a collection of endophytic root nodule bacteria. This work is among the first in-depth analyses of the role of PGP bacteria in the Mopane ecoregion, as well as in forests and woodlands in African savanna ecosystems.

## 2. Materials and Methods

### 2.1. Site Description

Together with the Kruger National Park (South Africa) and the Gonarezhou National Park (Zimbabwe), the Limpopo National Park (LNP, Mozambique) belongs to the Great Limpopo Transfrontier Park (GLTP) [21], a transboundary community-based initiative for biodiversity conservation, peace, and socioeconomic empowerment [22]. The GLTP covers more than 35,000 km^2^, constituting an important biodiversity repository of mammals (146 species), reptiles (114 species), and birds (550 species) [23]. LNP (Figure S1a) is located between parallels 22°25′ S–24°10′ S and 31°18′ E–32°39′ E in the Gaza Province, covering a total area of ca. 1,000,000 ha [24]. The climate is subtropical, with wet summers and mild, dry winters. The mean annual temperature is 30 °C, increasing from south to north, but temperatures can reach as high as 40 °C during the months of November to February. The mean annual rainfall varies from 360 mm in the north to 500 mm in the south [25]. The work was conducted in the three dominant landscapes of the LNP: Calcrete (C), Lebombo North (LN), and Nwambia Sandveld (NS) [24]. The first landscape (C) occupies 38.8% of the park and is characterized by sedimentary footslopes and ravines with shallow and calcareous soils. LN corresponds to 3.5% of LNP, being composed of stones and rocks with shallow soils derived from rhyolites. NS covers 44% of the total area, being composed of sandy substrates [24].

### 2.2. Sampling

Sampling sites were selected during field trips to LNP and through map analysis based on accessibility, fire frequency, and the three dominant landscapes (C, LN, and NS) [15] (Table 1; Figure S1a). In each sampling site, eight circular 40-m-diameter plots arranged in two contiguous parallel transects (4 plots per transect) were surveyed (Figure S1b). Rhizosphere soil samples (i.e., 3 landscapes × 2 fire regimes × 8 plots × 10 individuals) were collected 10 cm from the base of each focal tree at a depth of 20–30 cm with a plated soil probe. Samples from each experimental plot were pooled (Table 1), transferred to zip-lock bags, and kept at −80 °C until DNA extraction.

### 2.3. DNA Extraction and Illumina Sequencing of the 16S rRNA Gene

DNA was extracted from 100 mg soil samples using the DNeasy PowerSoil Kit (Qiagen, Germantown, MD, USA) following the manufacturer’s instructions. Briefly, 100 mg soil samples were added to PowerBead tubes, mixed with 60 μL of solution C1, vortexed for ca. 10 min, and centrifuged at 10,000× *g* for 30 s. For cell lysis, 250 μL of solution C2 was added to the supernatant, vortexed, and incubated at –8 °C for 5 min. After centrifugation at 10,000× *g* for 60 s, the supernatant was mixed with 200 μL of solution C3 and vortexed briefly. The mixture was incubated at −8 °C for 5 min to remove inhibitors and centrifuged at 10,000× *g* for 60 s. The supernatant was then mixed with 1.6 vol. of solution C4 to bind DNA, loaded onto an MB Spin Column, and centrifuged at 10,000× *g* for 60 s. The flow-through was discarded and 500 μL of solution C5 was added onto the column to wash the DNA twice at 10,000× *g* for 60 s. Finally, the DNA was eluted with 50 μL of solution C6. Library preparation and Illumina MiSeq paired-end 300 bp run was performed through services acquisition (Macrogen, Seul, South Korea). Briefly, DNA was first quantified by PicoGreen (Invitrogen, Waltham, MA, USA) using Victor 3 fluorometry and the quality was determined on a 2100 Bioanalyzer (Agilent technologies, Santa Clara, CA, USA). To analyze the bacterial composition of the samples, the V3-V4 region of the 16S rRNA gene was amplified by polymerase chain reaction (PCR) under the following conditions: initial denaturation at 98 °C for 2 min, followed by 35 cycles of denaturation at 95 °C for 30 s, primer annealing at 53 °C for 40 s, extension at 72 °C for 1 min, and a final extension phase at 72 °C for 5 min. Specific primers V3/V4 341F (5′-CCT ACG GGG NGG CWG CAG-3′) and 805R (5′-GAC TAC HVG GGT ATC TAA TCC-3′) and their adapters at the 5’ and 3’ ends of the DNA fragments were used [26]. The sizes and amounts of PCR-enriched fragments were verified on a 2100 Bioanalyzer (Agilent technologies, Santa Clara, CA, USA). The libraries were normalized before sequencing with the Illumina MiSeq system (Illumina, San Diego, CA, USA).

### 2.4. Sequence Data Analysis

Raw sequencing data were treated with MOTHUR v1.33 [27] following the pipeline previously used by Montagna et al. [28]. After making contigs using the *make.contigs* command with default settings, bacterial 16S rRNA raw sequences were filtered based on the following specifications: Phred score > 30, minimum fragment length of 250 bp, absence of ambiguous nucleotides (which are sometimes generated during contig formation when the delta between quality scores of a mismatched base is lower than 6), maximum 10 bp-long homopolymers, and maximum primer mismatch of 1 bp. Sequences were clustered based on 100% similarity and singletons were removed from the data set. Potential chimeric sequences were identified de novo and removed using the UCHIME algorithm [29]. Sequences were then clustered de novo into operational taxonomic units (OTUs) at 97% similarity using the open-source VSEARCH tool [26]. Although the 16S rRNA does not evolve uniformly along its length, the 3% dissimilarity cut-off was often chosen to distinguish bacterial species [30]. Pruning of OTUs with low number of sequences (<5) was carried out on a per-sample basis, as an OTU that is common in one sample may occur as a low-abundance contaminant in others due to cross-contamination [31]. The most abundant sequence of each OTU was selected as representative. Taxonomy was assigned through a search for similar sequences conducted with BLAST v2.2.29 [32] against the 13_5 release of the Greengenes online database [33]. The 16S rRNA gene database (http://greengenes.lbl.gov) is a curated database that addresses limitations of public repositories such as NCBI by providing chimera screening, standard alignment, and taxonomic classification using multiple published taxonomies. It is one of the two databases (together with SILVA) mostly used and integrated in MOTHUR or QIIME pipelines [34].

At this point, rarefaction curves were computed to evaluate the sequencing efforts provided. As a normalization step to reduce bias associated with different sequencing depths, all samples were subsampled down to the size of the smallest sample. OTU counts were then corrected according to the 16S rRNA copy number using the bioinformatics software package PICRUST [35] and re-subsampled at ~90% of the minimum corrected sample size. New OTUs were clustered according to their Greengenes ID for further statistical analyses.

All analyses were done using R v3.2.0, namely the R packages *vegan* [36] and *indicspecies* [37]. The OTU count was normalized on a per-sample basis using a square root transformation and Wisconsin double standardization in R v3.2.0 [38] prior to statistical analysis. As a final step of error correction, rare OTUs (<0.001% of total count) were removed from the OTU table. A Kruskal–Wallis or a Mann–Whitney test was performed on these indices, as well as on the number of OTUs to determine the effects of soil type and fire regime. A PERMANOVA test (999 permutations) based on a Bray–Curtis distance matrix calculated on the normalized OTU counts was carried out to determine the effect of fire incidence on the bacterial community structure. The heterogeneity of the communities was tested with a beta dispersion analysis. A non-metric multidimensional scaling (NMDS) biplot based on the previously computed Bray–Curtis distance matrix was constructed to graphically assess the differences in the community compositions in response to fire incidence. Beta diversity was also estimated by computing a phylogenetic tree based on unweighted UniFrac distances [39], which describe the dissimilarities between samples by assessing the evolutionary distances, taking into account differences in taxa abundance. The UniFrac distance matrix was visualized using principal coordinates analysis (PCoA). The unweighted pair group method with arithmetic mean (UPGMA) for clustering of samples was performed as an alternative hierarchical clustering method to interpret the UniFrac distance matrix. Both analyses were performed in QIIME (http://qiime.org/scripts).

### 2.5. Isolation and Characterization of Bacteria from Soils

Six composite soil samples from the three landscapes (C, LN, and NS) subjected to high and low fire frequencies were used for the isolation of soil bacteria. Aliquots of the soil samples were suspended in distilled water, diluted to 1:5 and 1:10, and plated on yeast mannitol (YM) [40] and Luria–Bertani (LB) [41] agars. Plates were incubated at 27 °C for 72 h. Single colonies were selected based on their morphology and repeatedly transferred to fresh agar plates until purified. Isolated bacteria were routinely grown in YM or tryptone–yeast (TY) [42] broth. Stock cultures were preserved in YM agar slants at 4 °C and in TY broth with 45% glycerol at −20 °C.

Soil bacterial isolates were evaluated for in vitro activities related to the promotion of plant growth using the same procedures as described previously [43]. The ability to growth in nitrogen-free media was used as a preliminary indicator of diazotrophy. Bacteria were isolated using the protocol of Döbereiner et al. [44] for the evaluation of microaerophilic growth in the absence of nitrogen [43]. Growth was evaluated by the formation of a subsurface pellicle. For evaluation of aerobic growth in the absence of nitrogen, the cell suspensions were streaked onto Burk’s N-free agar plates [45] and growth was evaluated after incubation for 5–7 days at 30 °C. 

The production of auxins was evaluated according to Asghar et al. [46]. Absorbance was measured at 535 nm after 30 min incubation at room temperature. Auxin levels were estimated as indole acetic acid (IAA) equivalents, using an IAA standard curve for calibration.

The ability to solubilize mineral phosphate was evaluated in yeast extract–dextrose (YED) agar plates supplemented with 5 g L^−1^ Ca_3_(PO_4_)_2_ [47]. The formation of clearance haloes around colonies was considered as indicative of phosphate solubilization.

Siderophore production was determined by the chrome azurol (CAS) assay [48], as modified by Pérez-Miranda et al. [49]. The formation of orange haloes around colonies was considered as indicative of siderophore production.

### 2.6. Isolation and Characterization of Root Nodule Bacteria Using a Host Legume (Vigna Unguiculata)

*Vigna unguiculata* (cowpea) plants from a local landrace (Portugal) were used as a trap host for rhizobia bacteria. Cowpea seeds were washed with ethanol for 1 min and surface-sterilized according to Somasegaran and Hoben [50]. After 1–2 h in sterilized water, seeds were transferred to 0.8% *w*/*v* water agar plates for 2 to 4 days until germination. Seedlings were transferred to jars containing inert sand and 50 mL of N-free Jensen plant nutrient medium [51]. For inoculation, 1 ml suspension of approximately 10^9^ bacterial cells in liquid Jensen medium (¼ diluted) was applied on the roots of each seedling. Additionally, a N-control (TN) with 2 mL of 1.75% KNO_3_ and a non-inoculated, N-free control (T0) supplemented with 1 mL of liquid Jensen medium (¼ diluted) were also prepared. Four replicates were performed for each selected strain or soil type. Plants were grown in an environmental chamber under light- and temperature-controlled conditions (16/8 h photoperiod and 23/18 °C) for the first 4 weeks and in a greenhouse for the last 7 weeks. Plants were watered with liquid Jensen medium once a week. The presence of nodules and the nodulation phenotype were examined after 11 weeks of plant growth. The dry weights of shoots were calculated after plant drying at 80 °C for 2 days and used to determine the index of symbiotic effectiveness (Es) according to the formula described by Ferreira and Marques [52]: Es (%) = (Xs − XT0/XTN − XT0) × 100, where Xs represents the mean dry weight of inoculated shoots; XTN represents the mean dry weight of plants with nitrogen control; XT0 represents the mean dry weight of uninoculated plants.

Bacteria were isolated from root nodules of *V. unguiculata* plants inoculated with the soil samples samples after 11 weeks of growth. Root nodules were surface-sterilized following the methodology described by Somasegaran and Hoben [50]. Nodules were individually crushed and a droplet of the nodule suspension was streaked on a yeast mannitol agar (YMA) plate containing Congo red [40]. Plates were incubated at 28 °C in the dark and observed for bacterial growth for 10 days. Isolate purity was checked by examining the colony morphology and Congo red absorption. Subculturing was conducted when more than one type of colony was present. A total of 23 isolates were stored at 4 °C until further genetic characterization through 16S rRNA gene sequencing (see Section 2.7).

### 2.7. Identification of Isolated Bacteria by 16S rRNA Gene Sequence Analysis

DNA from each purified isolate from both soils and trap nodules was extracted with the ExtractMe DNA Bacteria Kit (DNAGDANSK, Gdansk, Poland) using the protocol provided by the manufacturer. PCR amplification of the 16S rRNA region was carried out using the rD1 (5′-AAG GAG GTC ATC CAG CC-3′) and fD1 (5′-AGA GTT TGA TCC TGG CTC AG-3′) primers, following the protocol described by Weisburg et al. [53]. Briefly, PCRs were performed in 100 µL mixtures containing 0.5 µM of each primer, 2.5 U Taq DNA polymerase (Thermo Scientific, Whaltam, MA, USA), 200 µM dNTP, and 1.5 mM MgCl_2_ in 1x buffer (75 mM Tris–HCl pH 8.8 at 25 °C; 20 mM (NH_4_)2SO_4_; 0.01% (*v*/*v*) Tween 20). The PCR program consisted of 1 cycle at 95 °C for 3 min followed by 35 cycles at 94 °C for 1 min, 55 °C for 1 min, 72 °C for 2 min, and a final extension at 72 °C for 3 min. PCR products were resolved by agarose gel electrophoresis and purified with the GeneJet PCR purification kit (Thermo Scientific, Porto Salvo, Portugal). Sequencing was carried out at StabVida (Caparica, Portugal) and assembled after trimming using Geneious v10.2.4 (http://www.geneious.com) [54]. Assembled sequences were identified using the BLAST algorithm against the NCBI database [55,56]. 

## 3. Results and Discussion

### 3.1. Bacterial Diversity in Mopane Soils

The metabarcoding libraries yielded a total of 1,618,659 raw reads, with 397,253,396 bases sequenced. Statistics results of the raw data are presented in the Supporting Information Table S1. After the removal of low-quality bases, chimeras, contaminants, and rare sequence types, as well as following the normalization steps described in the materials and methods, a total of 4505 OTUs (Table 2) were obtained. Most rarefaction curves were able to reach the asymptote with a smaller number of sequences than the subsampling size, suggesting that the sequencing effort and the subsampling size were appropriate (Figure S2).

**Figure 1:**
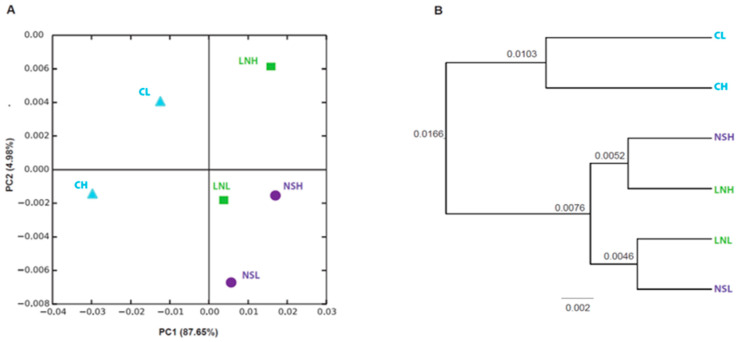
Beta diversity of mopane soil samples based on unweighted UniFrac distances. (**A**) Two-dimensional unweighted PCoA plot showing the separation of each sample type. (**B**) UPGMA tree showing the phylogenetic relationships between samples. C: Calcrete; LN: Lebombo North; NS: Nwambia Sandveld; H: high fire frequency; L: low fire frequency.

Independently of the plant species, landscape, or fire regime, no significant differences were found in the number of OTUs or in the values of the alpha diversity indices (Table 2). In accordance, the non-metric multidimensional analysis (NMDS) used to estimate the beta diversity index also showed no significant differences in bacterial community distribution across the three variables (Figure S3). Despite this, some spatial segregation was found in the PCoA plot (Figure 1A), as CL and CH samples were segregated from the remaining samples, a pattern also found in the UPGMA tree, although branches had very low support values (Figure 1B). The remaining samples were grouped in the same quadrant of the PCoA plot, except for LNH, while the UPGMA tree formed two groups for NS and LN samples related to the fire regime (Figure 1A).

Indeed, the impacts of forest wildfires on the soil microbiota are often associated with heat-induced mortality of microorganisms [57,58,59]. For example, while fire had a negative effect on the abundance of soil bacteria in the rocky mountain forest soils of Colorado (USA) [60], the opposite was observed in a temperate pine forest from Turkey and in a Mediterranean dense shrubland dominated by *Rosmarinus officinalis* [61,62] On the other hand, Hamman et al. [63] did not find considerable changes between bacterial communities from low- and high-severity burned sites (14 months post-fire). Thus, the impact of fire in soil microbial communities depends on the conjugation of several environmental and biogeochemical factors, such as temperature, soil texture, pH, and mineral and water contents [59,63,64]. According to our data, no major differences in bacterial OTU abundance were observed between the sampled sites independently of the soil type and fire frequency, indicating that soil bacteria can recover relatively fast [63]. In fact, wildfires are a major driver of biodiversity dynamics in the savannas, and many trees and shrubs in these ecosystems are well adapted to fire-prone environments through the development of fire tolerance and recovery strategies [16]. For example, in *Colophospermum mopane*, the presence of calcium oxalate crystals that promote the accumulation of CO_2_ to retard fire flames has been reported [14]. Therefore, it is possible that soil bacteria are also adapted to wildfires and that the changes induced by fire affect only the microbial biomass during a relatively short period (in our case less than 12 months, the date of the last fire in high-frequency sites). Future studies should investigate the most microbiologically relevant components in sites with different fire regimes and establish the timescale over which these effects persist. Examination of differential genomic changes in housekeeping genes or the existence of morphological adaptations in the microbial community would help to understand how they react and adapt to wildfires.

### 3.2. Taxonomic Composition of the Microbial Communities

**Figure 2:**
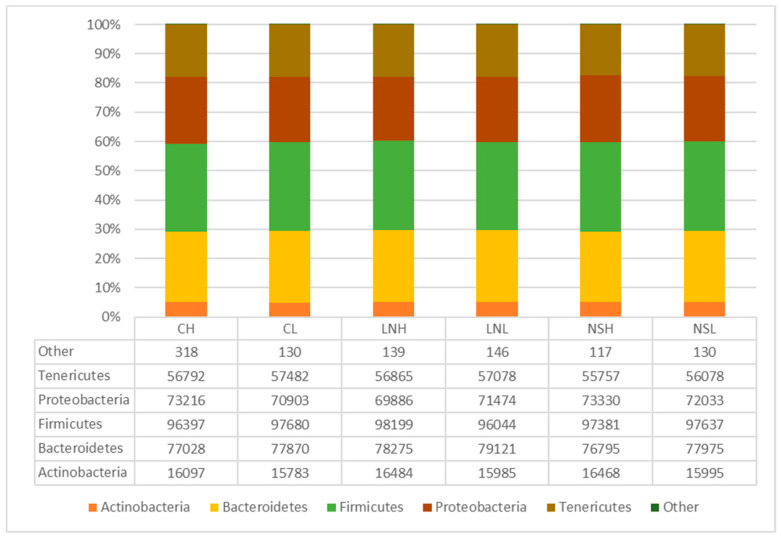
Relative abundance of bacterial phyla found in the sequences obtained from 16S rRNA gene-targeted Illumina sequencing. Population codes follow Table 1. The category “Other” (<1%) includes Acidobacteria, Armatimonadetes, Chloroflexi, Fusobacteria, Gemmatimonadetes, Lentisphaerae, Saccharibacteria, and Verrucomicrobia.

The taxonomic analysis of the bacterial composition showed that regardless of the plant species, landscape, and fire frequency, the phylum Firmicutes was most frequent (31%), followed by Bacteroidetes (25%), Proteobacteria (23%), Tenericutes (18%), and Actinobacteria (5%) (Figure 2). The most abundant orders were Bacteroidales (25%), Enterobacteriales (20%), RF39 (18%), Clostridiales (17%), and Erysipelotrichales (12%) (Figure S4), while the most abundant families were Enterobacteriaceae (24%), Erysipelotrichaceae (15%), Corynebacteriaceae (10%), and Coriobacteriaceae (9%) (Figure S5).

**Figure 3:**
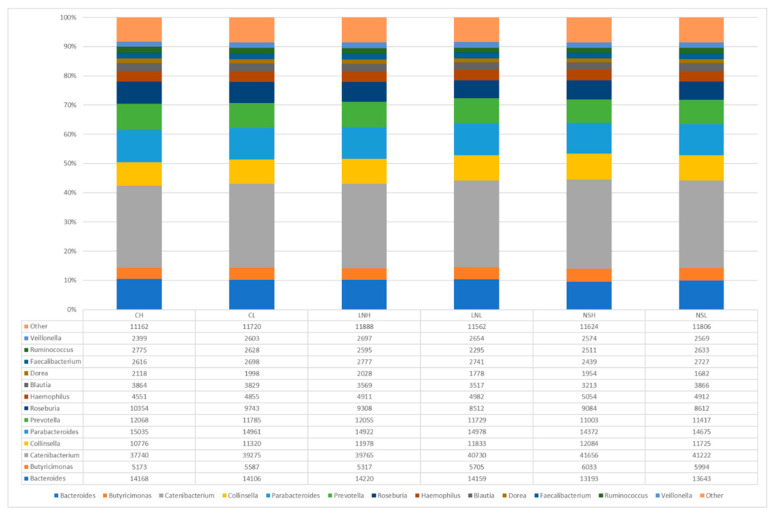
Relative abundance of bacterial genera found in the sequences obtained from 16S rRNA gene-targeted Illumina sequencing. Population codes follow Figure 1. The category “Other” (<4%) includes Haemophilus, Blautia, Ruminococcus, Faecalibacterium, Veillonella, and Dorea.

Regarding the genus, *Catenibacterium* was the most abundant one found in all sites (30%), followed by *Parabacteroides* (11%), *Bacteroides* (10%), *Prevotella* (9%), *Collinsella* (8%), *Roseburia* (7%), and *Butyricimonas* (4%) (Figure 3). Among these groups, several bacteria can cope with high temperatures, either by producing resistance structures such as endospores (Firmicutes) or spores (Actinobacteria), or because of their thick cell walls [65]. Indeed, several studies have found an enrichment of these groups after fire, particularly Firmicutes and Actinobacteria, as a consequence of their resistance to heat (e.g., [66,67])—in the case of Actinobacteria this is also linked to the ability to colonize post-fire environments [68]. 

The Limpopo National Park (LNP) where this work was developed contains 146 mammal species, including large grazing and browsing mammal species such as elephants (*Loxodonta africana*), buffalos (*Syncerus caffer*), giraffes (*Giraffa camelopardalis*), zebras (*Equus burchelli*), and impalas (*Aepyceros melampus*), which are important ecosystem drivers (along with rainfall, fire, and soil). Thus, the presence of *Catenibacterium* (and to a lower extent some of other Firmicutes) is most likely associated with the intestinal microbiota of these mammal species [69,70,71,72]. However, we should point out that *Catenibacterium* has also been found in soils from oil palm plantations [73] and maritime soils of the southern Antarctic [74], highlighting the need for further studies concerning the ecosystem functions of these microbes.

Because the metagenome analysis of LNP soils did not allow discrimination of most bacterial species (Table S2), we proceeded with the characterization of bacteria directly isolated from soil samples.

### 3.3. Taxonomic Analysis of Bacteria Isolated from Soils

Sixteen bacterial isolates were recovered from soils collected in the three landscapes of LNP, including six isolates from Calcrete (*C. mopane*), seven from Lebombo North (*C. mopane*), and three from Nwambia Sandveld (*Combretum apiculatum*) (Table 3). The isolated bacteria varied according to the landscape, fire regime, and plant species. Most isolates (13 out of 16) were assigned to the phylum Proteobacteria (Table 3).

Alpha Proteobacteria were represented by one isolate from Calcrete (CH4) and assigned as *Phyllobacterium myrsinacearum* (Figure S6). The genus *Phyllobacterium* (order *Rhizobiales*) contains many plant-associated bacteria that have been found in a variety of habitats, including the phyllosphere, endosphere, and rhizosphere of diverse plants, as well as in root nodules of legumes and leaf nodules of tropical ornamental plants [75].

Within beta Proteobacteria, two isolates from Calcrete (CL2) and Nwambia Sandveld (NSL2) were assigned, respectively, to the large group of *Burkholderia* s.l. (*Caballeronia concitans*, formerly *Burkholderia concitans*) [76] and *Paraburkholderia phenoliruptrix* (Table 3). *Caballeronia concitans* was assigned to the *Burkholderia glathei*-like group, which forms a phylogenetically divergent clade from other species belonging to the genus *Burkholderia*, including species with high functional diversity, mostly isolated from environmental samples. *Paraburkholderia phenoliruptrix* is reported to harbor symbiotic diazotrophic strains that are effective in fixing nitrogen in association with *Mimosoidea* [77]. 

Regarding gamma Proteobacteria, five isolates originated from rocky soils in Lebombo North were closely related to species of the fluorescens pseudomonads complex (*Pseudomonas azotoformans*, LNH1, LNL2, LNL4, *Pseudomonas synxantha*, LNL3, and *Pseudomonas gessardii*, LNL5). These organisms display a wide range of plant-beneficial traits, including direct stimulation of growth, mobilization of nutrients, and protection against diseases through the production of siderophores and other molecules with antimicrobial activity [43,78,79,80]. Two other isolates from Lebombo North (LNL1, LNH3) and one isolate from Calcrete (CH3) were assigned to *Pantoea agglomerans* (Table 3), a well-known plant-associated bacterium that has also been linked to opportunistic infections in humans [81]. Two isolates from Nwambia Sandveld (NSH1, NSH2) were assigned to *Stenotrophomonas maltophilia*, an environmental species present in several habitats, including rhizospheric soil, but also associated with respiratory tract infections in humans, being considered an emerging multidrug-resistant opportunistic pathogen of global dispersion [82].

Finally, the three remaining isolates were obtained from Calcrete (CL1, CL3, and CH2) and assigned to the genus *Bacillus* sp. (Table 3). The isolation of *Bacillus* from the roots of mopane trees has already been reported in the Kunene region of Namibia [20]. Together with *Acetobacter*, *Azotobacter*, and *Pseudomonas*, *Bacillus* species are among the most predominant plant-growth-promoting bacteria [83,84]. However, no specific *Bacillus* were assigned from our blasts to the NCBI database, which indicates that further studies are necessary to discriminate the species of *Bacillus* present in mopane soils.

### 3.4. In Vitro Activities Related to PGPB

Bacteria isolated from soils were also screened for the presence of in vitro activities related to the promotion of plant growth. Ten isolates from Calcrete (C) and Lebombo North (LN) were able to grow aerobically in nitrogen-free medium (Table 4). One isolate from (LN), *P. azotoformans* (LNL4), and two from Nwambia Sandvelt (NS), *S. maltophilia* (NSH1 and NSH2), were also able to grow in microaerophilic conditions in the absence of added nitrogen to the culture medium. These observations suggest that such isolates are nitrogen fixers. However, since no further evaluations for diazotrophy have been made (e.g., search for *nifH* gene sequences and measurement of nitrogenase activity), the possibility that these bacteria could grow on trace amounts of combined nitrogen from the medium or the atmosphere cannot be ruled out, thus possibly making them nitrogen scavengers instead of true nitrogen fixers [85].

Solubilization of tricalcium phosphate was observed in isolates assigned to *Bacillus* sp. (CH2 and CL3), *P. azotoformans* (LNH1) and *P. agglomerans* (LNH3). Because only a small fraction of phosphorus in soils is available to plants, strategies to improve phosphorus availability may contribute significantly to plant phosphorus nutrition and growth [86]. The ability of rhizospheric bacteria to solubilize soil-immobilized phosphates may represent an effective way of supplying phosphorus to plants.

Production of siderophores (high-affinity iron-chelating molecules) was detected in several isolates, including *P. phenoliruptrix* (NSL2), *Bacillus* sp. (CL1), *P. agglomerans* (LNH3, LNL1, and LNL2), and *Pseudomonas* spp. (LNH1, LNL3, LNL4, and LNL5). Interestingly, all seven isolates originating from LN (*Pseudomonas* spp. and *P. agglomerans*) were able to produce siderophores. The ability to produce siderophores may represent a competitive advantage for survival in iron-limited environments [79]. On the other hand, scavenging of iron from the rhizosphere may result in the exclusion of certain fungal phytopathogens by restricting their growth due to the lack of iron [87]. In this sense, the production of siderophores by the bacterial isolates may indicate important potential as biocontrol agents against plant diseases. Besides iron, siderophores can also form stable complexes with other heavy metals, and thus siderophore-producing bacteria may have agricultural applications in contaminated soils [88].

The most significant production of the plant hormone indole-3-acetic acid (IAA) was observed in *P. agglomerans* (LNH3) originating from (LN). Besides acting in the enhancement of root proliferation and stimulation of plant growth, the bacterial production of auxins may also be involved in the interaction of bacteria with plants as part of their colonization strategy, as well as in the circumvention of basal plant defense mechanisms [89]. The synthesis of high amounts of IAA from tryptophan was previously observed in a phylogenetic relative of *P. agglomerans* isolated from annual ryegrass [43]. In addition to IAA production, isolate LNH3 obtained in this work was also able to perform several other activities related to the promotion of plant growth, such as the production of siderophores, the solubilization of mineral phosphate, and the ability to grow in nitrogen-limiting conditions. Other isolates, e.g., *Pseudomonas* spp. (LNH1 and LNL3), were also able to accomplish multiple activities. These multifunctional bacteria have potential to be used as plant inoculants in different field situations, where they could act as phytostimulants, biofertilizers, or biocontrol agents, covering a range of agricultural and environmental applications. Undoubtedly, the results justify additional investigation to further assess in vivo the plant growth-promoting effects of the isolates, for instance using *C. mopane*, other model legumes (e.g., the tropical *Lotus japonicus* and the temperate *Medicago truncatula*), as well as non-legume plants such as *Arabidopsis thaliana*. It is interesting to note that soils from Lebombo North were the source of the most promising bacteria in terms of plant-beneficial activities, as opposed to Nwambia Sandveld, from which fewer and less active bacteria were isolated. Although the limited sample size does not allow a conclusion to be drawn on the relative abundance of putative PGPB in the three landscapes or for the different fire intensities, the results suggest that the rhizospheres of both *C. mopane* and *C. apiculatum* are potential sources of useful microbes that deserve a more detailed analysis.

### 3.5. Vigna unguiculata as a Trap for Rhizobia Bacteria

**Figure 4:**
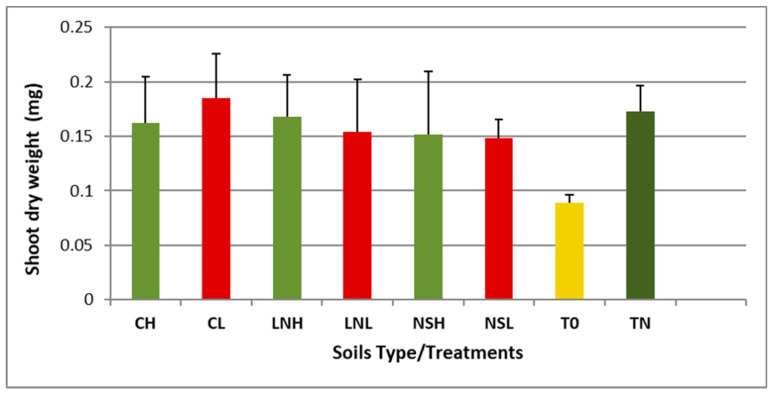
Dry weight of *Vigna unguiculata* plants inoculated with different soil samples. Population codes follow Figure 1. Uninoculated plants supplied either with nitrogen (TN) or without mineral N (T0) were also included. Values for shoot dry weight are the average of four replicates per soil type.

*Vigna unguiculata* (cowpea) is a well-studied tropical legume endemic from Africa [90], which is widely used as a trap plant due to its promiscuous symbiotic relationships regarding root nodule bacteria [91,92]. Therefore, we have used this crop as a trap host to isolate rhizobia species present in the rhizospheres of *C. mopane* and *C. apiculatum*. Inoculation of cowpea with any of the soil samples increased shoot dry weights to levels comparable to those of control plants supplied with nitrogen (Figure 4, Table S3), suggesting that the symbiosis with rhizobia bacteria was active. In fact, Es values were higher than 75%, indicating that the strains are highly effective in nitrogen fixation in all cases, except for the rhizobial natural population of NSL, which showed a moderate value (Es = 70%). These results are supported by the nodules’ phenotype (i.e., large and pink), suggesting that rhizobia actively fix nitrogen.

A total of 23 isolates were purified from *V. unguiculata* nodules. Most of these isolates were nitrogen-fixing bacteria belonging to the genus *Bradyrhizobium* (11 isolates; Table 5), while an additional one belonged to the genus *Rhizobium*. In total, 20 out of the 23 isolates were obtained from nodules of cowpea plants inoculated with soils of *C. mopane* (vs. 3 from nodules of plants inoculated with *C. apiculatum* soils). In fact, while *C. mopane* is a legume tree with primitive root nodules [93], *C. apiculatum* is neither a legume nor does it form symbiotic nitrogen-fixing root nodules. Thus, it was not surprising that soil samples from the rhizosphere of *C. mopane* were enriched in rhizobia isolates. On the other hand, although no previous studies were done in the rhizosphere of *C. mopane*, our results are in line with a pioneer study on the identification of nitrogen-fixing bacteria in the rhizospheres of the African legume trees, *Faidherbia albida* and *Albizia versicolor*, where *Bradyrhizobium* was the most predominant genus [94].

The remaining isolates obtained from *V. unguiculata* nodules were non-nodulating bacteria. Eight isolates were identified as *P. agglomerans.* These bacteria are related to several functions, among which is the production of indole acetic acid, and thus plant growth and health [43,89]. Lastly, three isolates were identified as *Azospirillum zeae* (LNH5.2)*, Pseudomonas nitroreducens* (LNH5.6), and *Cohnella rhizosphaerae* (LNL3.2). Curiously, several authors reported the presence of *Cohnella* spp. in soil samples [95], rhizosphere [96], and endophytic compartments, including plant root nodules [97]. Thus, our results suggest that endophytic bacteria coexist with rhizobial strains in nodules, playing supportive roles in plant growth and N_2_ fixation [98,99].

## 4. Concluding Remarks

In this study, we have isolated several bacterial species from the rhizosphere of *Colophospermum mopane* (and to a lower extent of *Combretum apiculatum*), which might play a role in adapting these species to wildfires. Although the global analysis did not show significant differences, the isolation of PGPB suggests that the rhizosphere bacteria are driven by the plant species, soil type, and fire regime, being potentially associated with a wide range of agricultural, environmental, and industrial applications. Several isolates were related to nitrogen or phosphorus nutrition (*Bacillus* sp., *Pantoea agglomerans*, *Caballeronia concitans*, *Pseudomonas* spp., and *Stenotrophomonas maltophilia*), as well as to biotic or abiotic stress tolerance (*Bacillus* sp., *P. agglomerans*, *Pseudomonas* spp., and *Paraburkholderia phenoliruptrix*). Moreover, recent studies have highlighted the importance of many of these species for bio-based solutions. For example, *Phyllobacterium myrsinacearum*, a metal-resistant bacterium, was reported as a promising soil bioremediator, particularly when associated with hyperaccumulator plants such as *Pteris vittata* [100] and *Sedum plumbizincicola* [101]. Additionally, *P. phenoliruptrix* is able to degrade recalcitrant xenobiotics [102], while *Pseudomonas synxantha* and *S. maltophili* are strong candidates for bioremediation of soil contaminated seawater [103] and chromium (Cr) [104] and estrogen [105], respectively. On the other hand, *Pseudomonas gessardii* has been indicated as a potential biocatalyst for the synthesis of bromohydrins [106] and for enzyme therapy [107]. Additionally, *P. agglomerans* is a promising multifunctional microorganism in biorefineries, namely in converting the hemicellulose fraction of plant biomass into valued-added products [108] and in producing plant-based food ingredients [109]. It is also a source of antibiotics against multidrug-resistant bacteria [110] and able to degrade waste [111]. Finally, *S. maltophilia* seems to be promising in medical therapy [112,113]. Regarding the nitrogen-fixing rhizobia isolated from root nodules of the trap host legume (i.e., cowpea inoculated with mopane rhizosphere soils), our results suggest that similarly to other African legume trees [94], *Bradyrhizobium* is the predominant genus in the rhizosphere of *C. mopane*. In conclusion, the rhizospheres of African savanna woodlands constitute untapped sources of microbial diversity with promising socioeconomic potential.

## Figures and Tables

**Table 1:** List of sampling sites in the three different landscapes studied, soil types, regimes of fire frequency (high vs. low), sample codes, and geographical coordinates. C: Calcrete; LN: Lebombo North; NS: Nwambia Sandveld. The letter H following a landscape code indicates high fire frequency, while L indicates low fire frequency [15].

Landscape	Soil Type	Fire Frequency	Last Fire	Code	Latitude	Longitude
C	Calcareous	High	1 year	CH	−23.6139	32.0242
			1 year		−23.3311	31.7095
		Low	7 years	CL	−23.3823	31.7697
			10 years		−23.6592	32.0485
LN	Rocky	High	1 year	LNH	−23.8177	31.7558
			1 year		−23.7192	31.7514
		Low	10 years	LNL	−23.6795	31.7329
			10 years		−23.7592	31.7548
NS	Sandy	High	1 year	NSH	−23.6321	32.1484
			1 year		−23.5752	32.0895
		Low	10 years	NSL	−23.5173	32.2516
			10 years		−23.5285	32.2213

**Table 2:** Bacterial diversity in soils. Lanscape codes follow Table 1. Statistical results are shown between soil types (H, Kruskal–Wallis test) and between fire regimes (U, Mann–Whitney test) and their respective significance values (P).

Landscape	Soil Type	Fire Frequency	OTU Number	Dominance D	Shannon H	Equitability J	Berger-Parker
C	Calcareous	Low	767	0.04023	4.085	0.6150	0.1215
		High	831	0.04116	4.078	0.6066	0.1275
LN	Rocky	Low	744	0.04109	4.067	0.6150	0.1230
		High	737	0.04004	4.087	0.6190	0.1192
NS	Sandy	Low	730	0.04157	4.065	0.6165	0.1256
		High	696	0.04282	4.040	0.6172	0.1292
Between Landscapes			H = 4.571 P = 0.102	H = 3.714 P = 0.156	H = 3.429 P = 0.180	H = 3.271 P = 0.257	H = 2.571 P = 0.276
Between fire regimes			U = 4.005 P = 0.827	U = 4.000 P = 0.810	U = 4.550 P = 0.850	U = 3.005 P = 0.617	U = 3.000 P = 0.613

**Table 3:** Taxonomical identification of soil isolates from LNP soils and related phylum according to the most related source organism deposited in GenBank. Population codes follow Table 1.

Landscape	Soil Type	Fire Frequency	Isolate	Most Related Source Organism	Phylum	GenBank Accession Number	Identity
C	Calcareous	High	CH2	*Bacillus* sp. strain QW2	Firmicutes	MT065751.1	100.0%
			CH3	*Pantoea agglomerans* strain DSM 3493	Proteobacteria	KY013009.1	98.7%
			CH4	*Phyllobacterium myrsinacearum* strain NBRC 100019	Proteobacteria	NR_113874.1	97.3%
		Low	CL1	*Bacillus* sp. strain PJA1.5	Firmicutes	MT275726.1	100.0%
			CL2	*Caballeronia concitans* strain LMG 29315	Proteobacteria	NR_145603.1	98.7%
			CL3	*Bacillus* sp. strain R43	Firmicutes	MT254891.1	100.0%
LN	Rocky	High	LNH1	*Pseudomonas azotoformans* strain LMG 21611	Proteobacteria	LT629702.1	99.9%
			LNH3	*Pantoea agglomerans* strain DSM 3493	Proteobacteria	MF289172.1	99.8%
		Low	LNL1	*Pantoea agglomerans* strain KB38	Proteobacteria	JF327464.1	97.9%
			LNL2	*Pseudomonas azotoformans* strain BG4	Proteobacteria	MK875666.1	99.9%
			LNL3	*Pseudomonas synxantha* strain HNR22	Proteobacteria	EU373390.1	98.9%
			LNL4	*Pseudomonas azotoformans* strain BG4	Proteobacteria	MK875666.1	99.6%
			LNL5	*Pseudomonas gessardii* strain 4G497	Proteobacteria	MG972901.1	99.8%
NS	Sandy	High	NSH1	*Stenotrophomonas maltophilia* strain PEG-305	Proteobacteria	CP040437.1	100%
			NSH2	*Stenotrophomonas maltophilia* strain PEG-305	Proteobacteria	CP040437.1	100%
		Low	NSL2	*Paraburkholderia phenoliruptrix* strain AC1100	Proteobacteria	NR_042901.1	100.0%

**Table 4:** In vitro plant-growth-promoting activities evaluated in soil isolates. Population codes follow Table 1.

Landscape	Soil Type	Fire Frequency	Isolate	Most Related Source Organism	Growth in N-Free Media ^a^		IAA Production ^b^	Phosphate Solubilization ^c^	Siderophore Production ^d^
					Aerobic	Microaerophilic	(µg mL^−1^)		
C	Calcareous	High	CH2	*Bacillus* sp. strain QW2	+	−	10.0 ± 0.1	+	−
			CH3	*Pantoea agglomerans* strain DSM 3493	+	−	<5.0	−	−
			CH4	*Phyllobacterium myrsinacearum* strain NBRC 100019	−	−	<5.0	−	−
		Low	CL1	*Bacillus* sp. strain PJA1.5	−	−	6.6 ± 0.9	−	+
			CL2	*Caballeronia concitans* strain LMG 29315	+	−	<5.0	−	−
			CL3	*Bacillus* sp. strain R43	+	−	9.2 ± 0.1	+	−
LN	Rocky	High	LNH1	*Pseudomonas azotoformans* strain LMG 21611	−	−	5.0 ± 0.3	+	+
			LNH3	*Pantoea agglomerans* strain DSM 3493	+	−	29.6 ± 0.5	+	+
		Low	LNL1	*Pantoea agglomerans* strain KB38	+	−	5.0 ± 0.1	−	+
			LNL2	*Pseudomonas azotoformans* strain BG4	+	−	<5.0	−	+
			LNL3	*Pseudomonas synxantha* strain HNR22	+	−	<5.0	+	+
			LNL4	*Pseudomonas azotoformans* strain BG4	+	+	<5.0	−	+
			LNL5	*Pseudomonas gessardii* strain 4G497	+	−	<5.0	−	+
NS	Sandy	High	NSH1	*Stenotrophomonas maltophilia* strain PEG-305	−	+	<5.0	−	−
			NSH2	*Stenotrophomonas maltophilia* strain PEG-305	−	+	<5.0	−	−
		Low	NSL2	*Paraburkholderia phenoliruptrix* strain AC1100	−	−	<5.0	−	+

^a^ Growth in Burk’s N-free (BNf) agar plates (aerobic) and semi-solid BNf medium (microaerophilic). ^b^ Average values determined in two replicate samples from cultures in Tryptone–Yeast Extract (TY) broth supplemented with 300 µg mL^−1^ tryptophan and incubated at 30 °C with shaking for 16 h. ^c^ Formation of a solubilization halo on Yeast Exctract-Dextrose (YED) agar supplemented with 5 g L^−1^ Ca(PO_4_)_2_. ^d^ Formation of an orange halo on TY agar as determined by the Chrome Azurol S (CAS) assay.

**Table 5:** Taxonomical identification of isolates from *Vigna unguiculata* root nodules inoculated with LPN soil samples, according to the most related source organism deposited in GenBank. Population codes follow Table 1.

Landscape	Soil Type	Fire Frequency	Isolate	Most Related 16S rRNA Gene Sequence (s)	Phylum	GenBank Accession Number	% Identity
C	Calcareous	High	CH1.1	*Bradyrhizobium sp.* strain C-145	Proteobacteria	MT229310.1	100.0%
			CH1.2	*Bradyrhizobium sp.* strain C-145	Proteobacteria	MT229310.1	99.85%
			CH1.3	*Pantoea agglomerans* strain CFSAN 047153	Proteobacteria	CP034469.1	99.86%
			CH1.4	*Pantoea agglomerans* strain UAEU 18	Proteobacteria	CP048033.1	98.94%
			CH1.5	*Pantoea agglomerans* strain CFSAN 047154	Proteobacteria	CP034474.1	99.79%
		Low	CL2.2	*Bradyrhizobium sp.* strain C-145	Proteobacteria	MT229310.1	100.0%
			CL2.3	*Bradyrhizobium sp.* strain C-145	Proteobacteria	CP022221.1	99.85%
			CL2.4	*Pantoea agglomerans* strain UAEU 18	Proteobacteria	CP048033.1	100.0%
			CL2.5	*Pantoea agglomerans* strain C410P1	Proteobacteria	CP016889.1	99.44%
			CL2.6	*Bradyrhizobium sp.* ORS 3257 isolate ORS3257	Proteobacteria	LS398110.1	100.0%
LB	Rocky	High	LNH5.1	*Pantoea agglomerans* strain C410P1	Proteobacteria	CP016889.1	99.02%
			LNH5.2	*Azospirillum zeae* strain N7	Proteobacteria	NR_043934.1	98.28%
			LNH5.3	*Bradyrhizobium sp.* strain C-145	Proteobacteria	CP022221.1	100%
			LNH5.5	*Rhizobium sp.* strain 11515TR	Proteobacteria	MK791683.1	99.49%
			LNH5.6	*Pseudomonas nitroreducens* strain HJ-3	Proteobacteria	MH324395.1	99.94%
		Low	LNL3.2	*Cohnella rhizosphaerae* strain 18JY42-3	Firmicutes	MH497628.1	97.38%
			LNL3.3	*Bradyrhizobium sp.* strain C-145	Proteobacteria	MT229310.1	99.93%
			LNL3.4	*Pantoea agglomerans* strain CFSAN 047,153	Proteobacteria	CP034469.1	99.33%
			LNL3.5	*Bradyrhizobium sp.* strain C-145	Proteobacteria	MT229310.1	97.37%
			LNL3.6	*Pantoea agglomerans* CFSAN 047153	Proteobacteria	CP034469.1	99.59%
NS	Sandy	High	NSH9.1	*Bradyrhizobium sp.* strain C-145	Proteobacteria	MT229310.1	99.91%
			NSH9.3	*Bradyrhizobium sp.* strain TUTMGGH52	Proteobacteria	CP030053.1	99.58%
			NSH9.4	*Bradyrhizobium sp.* strain C-145	Proteobacteria	MT229310.1	99.72%

## Supplementary Materials

The following are available online at https://www.mdpi.com/2076-2607/8/9/1291/s1, Table S1: Statistics of raw data of soil samples of Mopane woodlands. C: Calcrete; LN: Lebombo North; NS: Nwambia Sanveld; CH: Mopane rhizosphere on C with high fire frequency; CL: Mopane rhizosphere on C with low fire frequency; LNH: Mopane rhizosphere on LN with high fire frequency; LNL: Mopane rhizosphere on LN with low fire frequency; NSH: Combretum rhizosphere on NS with high fire frequency; NSL: Combretum rhizosphere on NS with low fire frequency; Table S2. Taxonomy assignment of representative sequences from each OTU from the six sampling areas (CH, CL, LNH, LNL, NSH, NSL) of our study. C: Calcrete; LN: Lebombo North; NS: Nwambia Sanveld; CH: Mopane rhizosphere on C with high fire frequency; CL: Mopane rhizosphere on C with low fire frequency; LNH: Mopane rhizosphere on LN with high fire frequency; LNL: Mopane rhizosphere on LN with low fire frequency; NSH: Combretum rhizosphere on NS with high fire frequency; NSL: Combretum rhizosphere on NS with low fire frequency; Table S3. Average dry weight (g) of Vigna unguiculata plants inoculated with different soil samples (mean ± SD). C: Calcrete; LN: Lebombo North; NS: Nwambia Sanveld; CH: Mopane rhizosphere on C with high fire frequency; CL: Mopane rhizosphere on C with low fire frequency; LNH: Mopane rhizosphere on LN with high fire frequency; LNL: Mopane rhizosphere on LN with low fire frequency; NSH: Combretum rhizosphere on NS with high fire frequency; NSL: Combretum rhizosphere on NS with low fire frequency. Uninoculated plants were also included supplied either with nitrogen (TN) or without mineral N (T0). Values of shoot dry weight are the average of four replicate/soil type. Es (%) was calculated as Es= (Xs − XT0/XTN − XT0) × 100, where Xs represents the mean dry weight of inoculated shoots; XTN the mean dry weight of plants with nitrogen control; XT0 the mean dry weight of uninoculated plants.
