# Supplementary material for: Mining the Microbiome of Key Species from African Savanna Woodlands: Potential for Soil Health Improvement and Plant Growth Promotion
Source: Microorganisms. 2020 Aug 24;8(9):1291. doi: 10.3390/microorganisms8091291 (PMC7563409; doi:10.3390/microorganisms8091291)
Supplement: Supplementary file 1 [file microorganisms-08-01291-s001.zip › microorganisms-843918 r 1 supplementary/Table S1 Statistics of raw data.docx]

**Table S1.** Statistics of raw data of soil samples of Mopane woodlands. C: Calcrete; LN: Lebombo North; NS: Nwambia Sanveld; CH: Mopane rhizosphere on C with high fire frequency; CL: Mopane rhizosphere on C with low fire frequency; LNH: Mopane rhizosphere on LN with high fire frequency; LNL: Mopane rhizosphere on LN with low fire frequency; NSH: Combretum rhizosphere on NS with high fire frequency; NSL: Combretum rhizosphere on NS with low fire frequency.

| Landscape | Soil type | Fire frequence | Total Bases | Read Count | N (%) | GC (%) | Q20 (%) | Q30 (%) |
| --- | --- | --- | --- | --- | --- | --- | --- | --- |
| C | Calcareous | High | 82937260 | 303797 | 0.0002 | 55.02 | 98.26 | 93.29 |
|  |  | Low | 70071123 | 287155 | 0.0003 | 53.84 | 99.2 | 96.15 |
| LN | Rocky | High | 58,363,861 | 246,374 | 0.0001 | 53.69 | 99.38 | 96.78 |
|  |  | Low | 65,207,919 | 274,602 | 0.0002 | 53.65 | 99.4 | 96.86 |
| NS | Sandy | High | 56,501,233 | 237,046 | 0.0003 | 53.57 | 99.43 | 96.93 |
|  |  | Low | 64,172,000 | 269,685 | 0.0003 | 53.59 | 99.42 | 96.88 |
|  | Total |  | **397,253,396** | **1,618,659** |  |  |  |  |
