# Supplementary material for: Mining the Microbiome of Key Species from African Savanna Woodlands: Potential for Soil Health Improvement and Plant Growth Promotion
Source: Microorganisms. 2020 Aug 24;8(9):1291. doi: 10.3390/microorganisms8091291 (PMC7563409; doi:10.3390/microorganisms8091291)
Supplement: Supplementary file 1 [file microorganisms-08-01291-s001.zip › microorganisms-843918 r 1 supplementary/Table S3 Symbiotic effect.docx]

**Table S3.** Average dry weight (g) of *Vigna unguiculata* plants inoculated with different soil samples (mean ±SD). C: Calcrete; LN: Lebombo North; NS: Nwambia Sanveld; CH: Mopane rhizosphere on C with high fire frequency; CL: Mopane rhizosphere on C with low fire frequency; LNH: Mopane rhizosphere on LN with high fire frequency; LNL: Mopane rhizosphere on LN with low fire frequency; NSH: Combretum rhizosphere on NS with high fire frequency; NSL: Combretum rhizosphere on NS with low fire frequency. Uninoculated plants were also included supplied either with nitrogen (TN) or without mineral N (T0). Values of shoot dry weight are the average of four replicate/soil type. Es (%) was calculated as Es= (Xs-XT0/XTN-XT0) x 100, where Xs represents the mean dry weight of inoculated shoots; XTN the mean dry weight of plants with nitrogen control; XT0 the mean dry weight of uninoculated plants

| **Landscape** | **Soil type** | **Fire frequence** | **Dry weight (g)** | **% Es** |
| --- | --- | --- | --- | --- |
| C | Calcareous | High | 0.162 ± 0.085 | 87.22 |
|  |  | Low | 0.184 ± 0.081 | 114.26 |
| LN | Rocky | High | 0.168 ± 0.076 | 94.28 |
|  |  | Low | 0.154 ± 0.094 | 78.05 |
| NS | Sandy | High | 0.151 ± 0.114 | 74.99 |
|  |  | Low | 0.148 ± 0.034 | 70.52 |
| T0 |  |  | 0.088 ± 0.014 | 0 |
| TN |  |  | 0.172 ± 0.047 | 100 |
